# Individual‐specific functional connectome biomarkers predict schizophrenia positive symptoms during adolescent brain maturation

**DOI:** 10.1002/hbm.25307

**Published:** 2020-12-02

**Authors:** Yun‐Shuang Fan, Liang Li, Yue Peng, Haoru Li, Jing Guo, Meiling Li, Siqi Yang, Meng Yao, Jingping Zhao, Hesheng Liu, Wei Liao, Xiaonan Guo, Shaoqiang Han, Qian Cui, Xujun Duan, Yong Xu, Yan Zhang, Huafu Chen

**Affiliations:** ^1^ The Clinical Hospital of Chengdu Brain Science Institute, MOE Key Lab for Neuroinformation, School of life Science and technology University of Electronic Science and Technology of China Chengdu China; ^2^ Department of Psychiatry The Second Affiliated Hospital of Xinxiang Medical University Xinxiang China; ^3^ Athinoula A. Martinos Center for Biomedical Imaging, Department of Radiology Massachusetts General Hospital, Harvard Medical School Charlestown Massachusetts USA; ^4^ Institute of Mental Health The Second Xiangya Hospital, Central South University Changsha China; ^5^ Department of Psychiatry First Hospital/First Clinical Medical College of Shanxi Medical University Taiyuan China

**Keywords:** adolescent, biomarker, functional connectome, individual level, schizophrenia

## Abstract

Even with an overarching functional dysconnectivity model of adolescent‐onset schizophrenia (AOS), there have been no functional connectome (FC) biomarkers identified for predicting patients' specific symptom domains. Adolescence is a period of dramatic brain maturation, with substantial interindividual variability in brain anatomy. However, existing group‐level hypotheses of AOS lack precision in terms of neuroanatomical boundaries. This study aimed to identify individual‐specific FC biomarkers associated with schizophrenic symptom manifestation during adolescent brain maturation. We used a reliable individual‐level cortical parcellation approach to map functional brain regions in each subject, that were then used to identify FC biomarkers for predicting dimension‐specific psychotic symptoms in 30 antipsychotic‐naïve first‐episode AOS patients (recruited sample of 39). Age‐related changes in biomarker expression were compared between these patients and 31 healthy controls. Moreover, 29 antipsychotic‐naïve first‐episode AOS patients (analyzed sample of 25) were recruited from another center to test the generalizability of the prediction model. Individual‐specific FC biomarkers could significantly and better predict AOS positive‐dimension symptoms with a relatively stronger generalizability than at the group level. Specifically, positive symptom domains were estimated based on connections between the frontoparietal control network (FPN) and salience network and within FPN. Consistent with the neurodevelopmental hypothesis of schizophrenia, the FPN–SN connection exhibited aberrant age‐associated alteration in AOS. The individual‐level findings reveal reproducible FPN‐based FC biomarkers associated with AOS positive symptom domains, and highlight the importance of accounting for individual variation in the study of adolescent‐onset disorders.

## Graphical abstract

Reliable frontoparietal control network‐based functional connectome biomarkers underlying positive symptom domains could be identified at the individual level, but not at the group level. Replication results indicated relatively better generalization ability of the prediction model by using individual‐based strategy compared with group‐level approach.

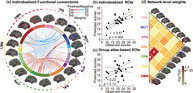

## 1 INTRODUCTION

Schizophrenia is a devastating psychiatric disorder with a typical onset in early adulthood. Adolescent‐onset schizophrenia (AOS), a relatively rare form, has more pronounced clinical symptomatology (Frazier et al., 2007) and tends to be more resistant to antipsychotic treatment, with worse prognosis (Hollis, 2000). Moreover, AOS is also characterized by more extensive neuropathological changes (Douaud et al., 2007). According to the overarching dysconnectivity hypothesis, the large‐scale organization of the brain—that is, the functional connectome (FC) plays an important role in the pathophysiology of schizophrenia (Li et al., 2018; Narr & Leaver, 2015; Woodward & Cascio, 2015). Patients with AOS have been reported to show FC abnormalities across widespread brain networks, including the default mode network (DMN) (Tang et al., 2013), limbic network (LMB) (White et al., 2008), frontoparietal control network (FPN) (Kyriakopoulos et al., 2012), and frontotemporal network (Yang et al., 2014). Dysconnectivity of these networks may scale with the severity of psychotic symptoms in patients (Tang et al., 2013; White et al., 2008). For example, aberrant LMB (White et al., 2008) and corticostriatal (Zheng et al., 2018) FC were separately reported to have implications for positive symptoms in AOS. Abnormally increased FC in DMN was also reported to be associated with positive symptoms (Tang et al., 2013). However, these dimensional findings have not cohered into a categorical set of brain‐based biomarkers that can be reproducibly used to predict AOS symptom burden.

To date, no reliable brain‐based biomarker that links neuroanatomical substrates to disease‐related behaviors has been identified for schizophrenic symptoms in adolescent patients. Brain‐based biomarker, as an indicator of neuronal function, can facilitate current diagnosis, prognosis, and treatment of psychotic illnesses (Tregellas, 2014; Yamada et al., 2017). Biomarkers for schizophrenic symptoms, which were supposed to be involved in patient's pathology, may be used to determine if therapeutic candidates evoke their targeted biological effects (Tregellas, 2014). Connectome‐based biomarkers are promising candidates for this purpose with recent advances in functional neuroimaging (Fan et al., 2020; Xiaonan Guo et al., 2020; Shaoqiang Han et al., 2020; Yamada et al., 2017). For instance, FC biomarkers have been identified to monitor social cognitive and neurocognitive performance in schizophrenia, which can further inform the treatment of cognitive deficits (Viviano et al., 2018). The lack of connectome‐based biomarkers for psychotic symptoms in AOS has thus far prevented progress in the diagnosis and treatment of this disorder.

Previous group‐level studies on AOS have been hampered by low precision in the mapping of functional cortical networks. Specifically, the brain undergoes dramatic alterations during adolescence, including cortical network reorganization and refinement (Cao, Huang, Peng, Dong, & He, 2016). Moreover, the brains of adolescents show greater interindividual variability than that of adults (Foulkes & Blakemore, 2018); this is especially true of the association cortex, which has been implicated in AOS pathology (Mueller et al., 2013). Inaccurate and indistinct neuroanatomical boundaries determined from group‐based approaches can prevent the delineation of brain connectome, and may obscure biologically important signals that can reveal brain–behavior associations in AOS (Gordon et al., 2017). Thus, accounting for the neuroanatomical variability among individuals is essential for establishing brain connectome and its relationship to symptomatology in AOS.

Recently, a reliable and reproducible cortical parcellation approach that account for individual heterogeneity in cortical functional anatomy has been developed by Wang et al. (Li et al., 2019; Wang et al., 2015). Based on an iterative functional network parcellation procedure, this individual‐level strategy maps cortical functional networks by localizing functional regions of interest (ROIs) in individual subjects. Dysconnectivities among these individual‐specific functional networks were reported in various psychiatric disorders including obsessive–compulsive disorder (Brennan et al., 2019), depression and AOS (Wang et al., 2018). Compared with group‐level analyses, FC based on individual‐level strategies had more robust predictive performance for whether cognitive abilities in healthy individuals (Li et al., 2019) or clinical symptoms in psychiatric illnesses (Brennan et al., 2019; Wang et al., 2018). Thus, using the individual‐specific parcellation approach may facilitate the discovery of FC biomarkers for psychotic symptoms in AOS.

To this end, the present study recruited 68 antipsychotic‐naïve first‐episode AOS patients including two independent replication cohorts. We employed functional connectivity analyses on resting‐state functional magnetic resonance imaging (MRI) signals of individual‐specific regions identified by the novel cortical functional network parcellation method, and applied a data‐driven prediction model to examine brain–behavior relationships. We speculated that the individual‐based strategy would increase statistical power by improving the specificity of functional signals in brain regions compared with the traditional group‐level approach. Specifically, we assumed that individual‐specific FC biomarkers could reproducibly and better predict specific schizophrenic symptom domains in the maturing adolescent brain than group atlas‐based biomarkers. According to the neurodevelopmental hypothesis of schizophrenia (Douaud et al., 2009), we also assumed that the predictive FC would show aberrant age‐related alterations in AOS compared with 31 healthy control (HC) subjects.

## 2 MATERIALS AND METHODS

### 2.1 Subjects

Thirty‐nine antipsychotic‐naïve first‐episode AOS patients and 31 sex‐ and age‐ matched HC subjects were recruited from outpatient treatment centers of the Second Affiliated Hospital of Xinxiang Medical University. Patients were diagnosed based on the consensus by two senior psychiatrists with more than 10 years of experience (Y. Z., and J. Z.) using the Structured Clinical Interview for Diagnostic and Statistical Manual of Mental Disorders, Fourth Edition, and the diagnosis was confirmed after a follow‐up of at least 6 months. Controls were recruited through media advertisements. Exclusion criteria for all subjects were as follows: neurological or other psychiatric diseases; current (within the last 12 months) substance use; neurological MRI anomalies; or any electronic or metal implants. The Positive and Negative Syndrome Scale (PANSS) was performed by the consensus of abovementioned two psychiatrists, which was used to assess the severity of psychotic symptoms in AOS patients. All participants were right‐handed, Han Chinese ethnicity, and aged from 12 to 18 years old. These subjects have previously participated in three hypothesis‐driven studies (Wang et al., 2017; Zheng et al., 2016; Zheng et al., 2018), which revealed disrupted frontoparietal functions in AOS. Our recent group‐level study examined the global efficiency of whole‐brain FC and reported disrupted large‐scale integration function (Li et al., 2018). However, previous group‐level studies were not enough to provide unbiased whole‐brain functional biomarkers for AOS symptoms. In the current study, three patients were excluded due to incomplete scanning, one patient due to excessive head motion (mean frame‐wise displacement, [FD] >0.2 mm), and five patients due to poor quality of intrasubject brain registration (cost >0.5). Ultimately, data for 30 AOS patients were used in the analysis. The demographic and clinical information is summarized in Table 1.

**TABLE 1:** Demographic and Clinical Characteristics

Characteristic	AOSs (*n* = 30)	HCs (*n* = 31)	Group comparisons	
			Statistic values	*p*‐Values
Sex (male/female)	15/15	13/18	0.40^a^	.53
Age (years)	15.10 ± 0.32	15.35 ± 0.28	0.59^b^	.55
Mean FD (mm)	0.02 ± 0.001	0.03 ± 0.004	383^c^	.11
PANSS scores				
Total scores	75.17 ± 2.00	—	—	—
General scores	34.13 ± 1.25	—	—	—
Positive scores	20.33 ± 1.08	—	—	—
Negative scores	20.70 ± 1.68	—	—	—

*Note*: Mean ± *SEM*.

Abbreviations: AOSs, adolescent‐onset schizophrenia patients; FD, frame‐wise displacement; HCs, healthy control subjects; PANSS, Positive and Negative Symptom Scale.

^a^ The *χ* ^2^ value for gender distribution was obtained by chi‐square test.

^b^ The *T* values were obtained by two‐sample *t* test.

^c^ The *U* values were obtained by Mann–Whitney tests.

This study was reviewed and approved by the Ethics Committee of the Department of Psychiatry at the Second Affiliated Hospital of Xinxiang Medical University and the Second Xiangya Hospital of Central South University, and written consent was obtained from all participants and their parents.

### 2.2 Data acquisition

Imaging data were collected using a 3 T MRI scanner (MAGNETOM Verio; Siemens, Germany). Patients were scanned before they were ever treated with antipsychotics. Specifically, they were scanned immediately after the first diagnosis was confirmed. Participants were instructed to stay awake with their eyes closed during the scan, and were asked if they had fallen asleep during the scanning at the end. Functional images were acquired as an echo‐planar imaging sequence with the following parameters: repetition time (TR) = 2,000 ms; echo time (TE) = 30 ms; matrix = 64 × 64, 33 axial slices; slice thickness = 4 mm, 0.6 mm gap; flip angle = 90°; field of view = 220 × 220 mm^2^; voxel size = 3.4375 × 3.4375 × 4 mm^3^; and 240 volumes. T1‐weighted anatomical images were acquired as a three‐dimensional fast‐spoiled gradient‐echo sequence with the following parameters: TR = 2,530 ms; TE = 2.43 ms; matrix = 256 × 256, 158 axial slices; slice thickness = 1.2 mm, no gap; flip angle = 7°; field of view = 256 × 256 mm^2^; and voxel size = 1 × 1 × 1 mm^3^.

### 2.3 Data preprocessing

Resting‐state functional images were preprocessed as previously described ^(Yeo et al.,^ ^2011^ ^)^. Briefly, the first four volumes were discarded; slice‐time and head motion (cut‐off <2 mm) were corrected with the FSL package; global mean signal intensity was normalized; a 0.01–0.08 Hz band‐pass temporal filter was applied; and head motion, ventricular, white matter, and cerebrospinal fluid signals were regressed out along with whole brain signal to improve the correction of motion‐related artifacts (Fan et al., 2019; Han et al., 2019; Yan et al., 2013). The mean FD was calculated for each participant. Subjects with the mean FD value exceeding the 0.2 mm were excluded from the analysis. Motion‐confounded time points were not censored as data scrubbing can increase connectivity estimates in specific regions (Guo et al., 2019; Zeng et al., 2014). However, head motion‐related functional connections (*p* < .01) among homologous individual‐specific ROIs were excluded in the subsequent prediction analysis (Wang et al., 2018). The contribution of head motion to the prediction results was further estimated by correlating mean FD and predicted scores for each subject. No correlation between mean FD and predicted scores (*r* = −.15, *p* = .44; See Figure S3 in Supplementary 3) indicated that head motion had no contribution to the results.

T1‐weighted anatomical images were preprocessed using the FreeSurfer v.5.3.0 software package (https://surfer.nmr.mgh.harvard.edu/). A validation analysis was added by normalizing the anatomical images into an adolescent template (http://www.bic.mni.mcgill.ca/ServicesAtlases/NIHPD-obj1) to exclude the influence of normalization template (see Supplementary 1 for detailed validation results). The structural and functional images were aligned by boundary‐based registration. Participants with the intrasubject registration cost exceeding the 0.5 were discarded. Functional images were registered to the FreeSurfer surface template; smoothed with a 6 mm full‐width half‐maximum smoothing kernel; and then downsampled to a mesh of 2,562 vertices in each hemisphere.

### 2.4 Identifying individual‐specific functional regions

The analytical procedure that was implemented for identification of individual‐specific functional ROIs has been previously described (Wang et al., 2018). Briefly, we used the iterative parcellation algorithm (Wang et al., 2015) to map 18 individual‐level cortical networks based on a group‐level functional network atlas (see Table S2 in Supplementary 3) (Wang et al., 2015), which was adapted from the original 17‐network atlas derived from 1,000 healthy subjects (Yeo et al., 2011). Specifically, individual‐level network boundaries were iteratively adjusted using the interindividual variability and signal‐to‐noise distributions. Based on the assumption that a group‐level ROI might roughly represent the center of the homologous ROIs across different individuals (Li et al., 2019), we used the group‐level ROI as the common reference. Thus, individual‐level cortical networks were segmented into discrete patches, and were matched to the 116 cortical ROIs extracted from the group‐level functional network atlas by using a clustering approach in FreeSurfer (mri_surfcluster). If a patch overlapped with a single or multiple ROIs in the atlas, it was labeled as the same ROI or split into multiple matched patches; however, if there was no overlap with an ROI, the patch was assigned to the nearest one (or labeled as “unrecognized”) if the mean distance between them was within a certain threshold (or exceed the threshold), which was selected as the mean distance between any two vertices in the nearest ROI. Finally, patches that matched the group‐level atlas‐based ROIs were labeled as the homologous ROIs in the individual. Functional connectivity analyses were performed on homologous ROIs across individuals to generate individual‐specific FC.

### 2.5 Predicting AOS symptoms

Based on FC among individual‐specific ROIs, the L2‐regularized and L2‐loss support vector machine for regression (SVR; using the default parameter C = 1) in the Library for Support Vector Machines toolbox was trained to predict the severity of AOS symptoms including positive and negative symptom scores, separately. Sex and head motion (mean FD) were included in the model as covariates. Age was not regressed out so that age‐related FC changes could be examined. The 10‐fold cross‐validation (CV) approach was applied to avoid biased estimates (Varoquaux et al., 2017). Specifically, the model was trained using randomly splitting 90% of the subjects and was used to estimate the symptom severity of the remaining subjects. Given the redundant feature sets, connections that were significantly correlated with symptoms (*p* < .01) were manually selected as training features in each CV to reduce redundancy and prevent over‐fitting. After repeating the procedure 10 times, predicted symptom scores were obtained for all subjects. The correlation coefficient between observed and predicted scores was calculated, which was used to evaluate the prediction performance. A permutation test (5,000 permutations) was performed by randomly reshuffling the observed clinical scores among the subjects to determine whether the correlation was simply due to chance. The entire SVR steps including the feature selection were rerun in each permutation.

The weight score of each feature in the SVR model was calculated to quantify the contribution of each cortical connection. Specifically, the score was computed by summing the times that the feature is not zero across all folds. If a connection was not selected out in one fold, then its contribution to this fold was set to zero. To summarize current results, these ROI‐ROI features were further grouped into network‐level connections according to seven well‐studied canonical networks, including visual network (VIS), sensorimotor network (MOT), attention network (ATN), salience network (SAL), FPN, LMB, and DMN. Statistical significance of the contribution of each between‐ or within‐network connection was estimated by comparing the weight values for each network‐level feature with the corresponding null distribution established by permutation tests. The Bonferroni‐corrected significance level was *p* < .05 divided by the number of network‐level connections (including 21 between‐network connections and seven within‐network connections).

### 2.6 Exploring age‐related alterations in predictive FC biomarkers

The neurodevelopmental hypothesis of schizophrenia (Douaud et al., 2009) posits that delayed and altered maturation of brain networks contributes to this disorder (Li et al., 2018; Zalesky et al., 2015). We therefore compared age‐related changes in predictive FC biomarkers between AOS patients and HC subjects. Specifically, we separately calculated correlations between age and connectivity values within the seven‐network model, which were obtained by averaging connectivity values (z values) of belonging ROI‐level connections, in the two groups. We used Shepherd's pi correlation to account for potential outliers and increase statistical power. The Bonferroni‐corrected significance level was *p* < .05 divided by the number of network‐level connections (including 21 between‐network connections and seven within‐network connections). The Fisher's z test was used to compare correlation coefficients of the two groups.

### 2.7 Validation analyses

To test the generalizability of the prediction model (Varoquaux et al., 2017), we recruited an independent replication cohort (29 age‐, sex‐, and psychotic symptom severity‐matched right‐handed antipsychotic‐naïve AOS patients) from outpatient treatment settings at the First Hospital of ShanXi Medical University (see Supplementary 2 for detailed participants' information and data acquisition). Data preprocessing of the replication cohort was identical with abovementioned preprocessing pipeline of the primary cohort. Two patients were excluded due to excessive head motion (mean FD > 0.2 mm) and two due to poor quality of intrasubject brain registration (cost >0.5). Ultimately, data for 25 AOS patients were used as a test set to compute predictive power of the model trained by all the primary data. Specifically, the SVR model used in the primary sample was applied without modification to the replication cohort, thus suggesting that the training features in the replication model were identified completely independently of the replication sample.

## 3 RESULTS

**FIGURE 1:**
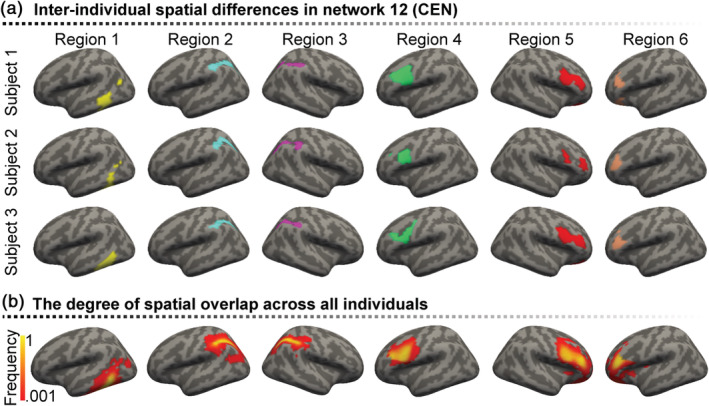
Interindividual spatial differences in six exemplary regions of interest (ROIs). (a) These cortical ROIs, which belong to the 12th network (i.e., the central executive network, CEN), demonstrate evident interindividual differences in size and position. (b) The frequency map of each vertex occurred in a certain ROI across all subjects was calculated to demonstrate the degree of interindividual spatial overlap

We identified 79 homologous ROIs for each subject, which were extracted from 18 individual‐specific cortical functional networks using an iterative parcellation algorithm. These ROIs were further grouped according to seven well‐studied canonical functional networks, including VIS, MOT, ATN, SAL, FPN, LMB, and DMN. From a visual perspective, the sizes and locations of the ROIs varied among individuals (Figure 1). Substantial interindividual variability in ROI size and position was demonstrated in Supplementary 4. Individual‐specific connectomes across these ROIs were evaluated and used to predict AOS symptoms in order to identify FC biomarkers based on the relationship between neuronal connectivity and behavior.

### 3.1 Individual‐level FC biomarkers predict specific symptom severity

**FIGURE 2:**
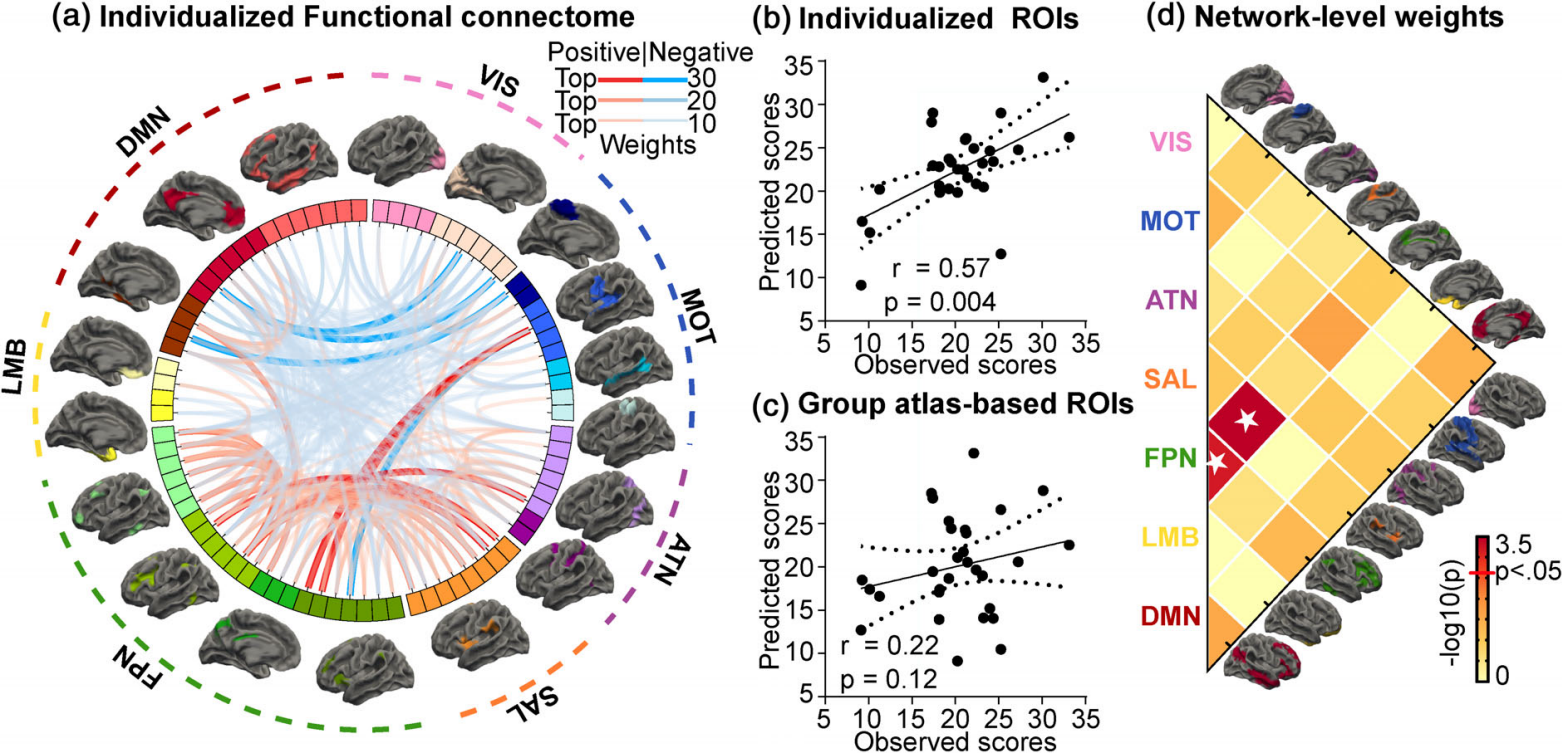
Individual‐level positive symptom prediction model. The individual‐specific functional connectome (FC) predicts positive symptom severity in adolescent‐onset schizophrenia (AOS). (a) Plot of 302 predictive connections among 79 homologous regions of interest (ROIs), which were extracted from 18 functional networks (shown outside the wheel) that are color‐coded according to seven well‐studied canonical networks, including the visual network (VIS), sensorimotor network (MOT), attention network (ATN), salience network (SAL), frontoparietal control network (FPN), limbic network (LMB), and the default model network (DMN). (b) Scatterplot illustrating the correlation (*r* = .57, *p* = .004; permutation test) between observed Positive and Negative Syndrome Scale (PANSS) positive scores and scores predicted by individual‐specific FC in AOS patients. (c) Group‐level atlas‐based ROIs were used to predict PANSS positive scores; this group‐level model could not predict the severity of AOS positive symptoms (*r* = .22, *p* = .12; permutation test). Solid line and dashed lines represent the best‐fit line and 95% confidence interval, respectively. (d) Statistical significance of predictive weight of each network‐level connection (color‐coded on the map) estimated by permutation test (with Bonferroni correction)

PANSS positive scores of AOS could be predicted by a set of functional connections (*r* = .57, *p* = .004; permutation test) (Figure 2b). From a visual perspective, individual‐specific connections that contributed most to the positive score prediction were mainly negative connections of the DMN and positive connections of the FPN (Figure 2a). The contribution of each network‐level connection to symptom prediction were quantified by summing weights of the included cortical ROI connections, and were further compared with corresponding random results obtained by permutation tests to estimate its statistical significance. Network‐level connections between FPN and SAL (*p* = .006; Bonferroni‐corrected) and within FPN (*p* = .02; Bonferroni‐corrected) had significant contribution in AOS patients (Figure 2d). Additionally, a group‐level functional network atlas was used to establish the connectome for comparing the predicted results based on individual‐ and group‐level ROIs. For the group atlas analysis, all steps were repeated including feature selection in order to avoid biases in favor of the individualized analysis. The group‐level atlas‐based FC was unable to predict PANSS positive scores in AOS patients (*r* = .22, *p* = .12; permutation test) (Figure 2c). Moreover, the correlation coefficient of individualized atlas‐based prediction model was significantly stronger (z = 2.72, *p* = .003; Steiger's z test) than that of group‐level model, indicating a better prediction power of the individual‐based strategy compared with group‐level approach. PANSS negative scores could not be predicted by either individual or group‐level atlas‐based FC. Additional exploratory analyses based on two PANSS five‐factor models (Lindenmayer, Bernstein‐Hyman, & Grochowski, 1994; Marder, Davis, & Chouinard, 1997; Wallwork, Fortgang, Hashimoto, Weinberger, & Dickinson, 2012) were performed, results of which supported our main results (see Supplementary 5 for detailed information).

### 3.2 Predictive FC biomarker of AOS shows aberrant age‐related alterations

**FIGURE 3:**
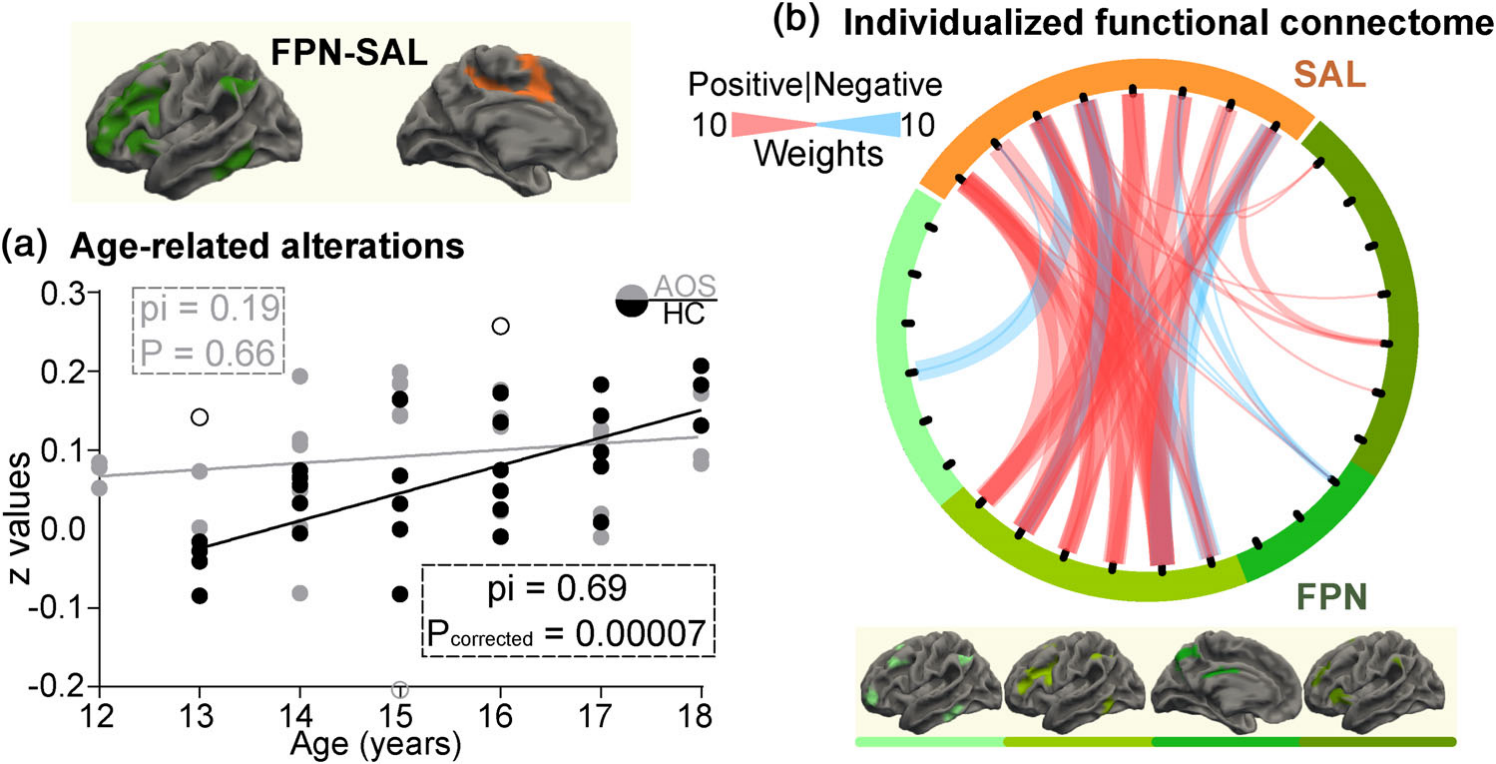
Age‐related alterations in individual‐specific functional connectome (FC) biomarker expression. In adolescent‐onset schizophrenia (AOS), network‐level connections between frontoparietal control network (FPN) and salience network (SAL) contributing the most to the prediction of Positive and Negative Syndrome Scale (PANSS) positive scores showed abnormal age‐related changes. (a) Scatterplot illustrating Shepherd's pi correlation (with Bonferroni correction) between age and FPN–SAL connectivity values estimated by averaging all included ROI connectivities in AOS (gray) and HC (black) groups. Filled circles were included in the correlation analyses, whereas open circles were excluded. The pi is Pearson's *r* value estimated by the remaining data, and the *p* is doubled to account for outlier removal. (b) Predictive ROI connections belonging to the FPN–SAL between‐network connection were plotted. Red and blue lines represent positive and negative ROI connections, respectively

To validate the neurodevelopmental hypothesis of schizophrenia, we separately calculated correlations between network‐level connectivity values and age in AOS patients and HC subjects. In network‐level connections that predicted positive scores, the significantly weighted FPN–SAL connection showed age‐related increases in the HC group (pi = 0.69, *p* = .002; Bonferroni‐corrected) (Figure 3A), while no such changes were observed in AOS patients (pi = 0.19, *P*_unc_ = 0.66). Moreover, the significantly different correlation coefficients between the two groups (z = 2.43, *p* = .02; Fisher's z test) indicated abnormal FC alterations as age increased in AOS. The predictive FPN–SAL connection mainly consisted of positive connections between SAL and a frontoparietal subnetwork—that is, the central executive network (CEN) (Figure 3b), which includes the dorsolateral prefrontal cortex (dlPFC), medial and lateral posterior prefrontal cortices and some parts of the intraparietal sulcus and posterior temporal gyrus) (Vincent, Kahn, Snyder, Raichle, & Buckner, 2008).

### 3.3 Predictive power of these FC biomarkers in another independent cohort

**FIGURE 4:**
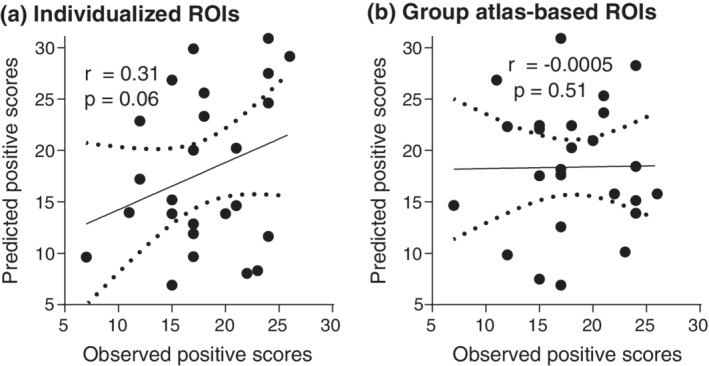
Predictive power of the primary positive symptom prediction model in another independent cohort. (a) Scatterplot illustrating the correlation (*r* = .31, *p* = .06; permutation test) between observed Positive and Negative Syndrome Scale (PANSS) positive scores and scores predicted by individual‐specific functional connectome (FC) in AOS patients. (b) FC based on group‐level atlas could not predict PANSS positive scores in AOS entirely (*r* = −.0005, *p* = .51; permutation test)

These FPN‐based Individual‐level FC biomarkers, which were trained by the primary data, could approximately predict PANSS positive scores of the replicated cohort (*r* = .31, *p* = .06; permutation test) (Figure 4a). On the contrary, the group‐level atlas‐based FC was unable to predict the scores entirely (*r* = −.0005, *p* = .51; permutation test) (Figure 4b). Moreover, the prediction power of the individualized prediction model tended to be better than that of the group‐level model (z = 1.49, *p* = .06; Steiger's z test). Thus, the replication results approximately supported the individualized ROI‐based prediction model, and indicated relatively better generalizability of the prediction model by using individual‐based strategy compared with group‐level approach.

## 4 DISCUSSION

In this study, we identified reproducible FC biomarkers among individual‐specific functional regions that could predict positive symptom domain in antipsychotic‐naïve first‐episode AOS. However, connectome based on group‐level atlas could not estimate AOS clinical symptoms in any specific dimensions. More robust predictive performance has been observed for individual as compared to group‐level strategies in multiple psychiatric disorders, including depression, AOS (Wang et al., 2018), and obsessive–compulsive disorder (Brennan et al., 2019). Moreover, recent study of AOS consistently indicated close relation between positive symptoms and FPN‐associated between‐network or within‐network connectivities, supporting the neurobiological continuity between AOS and its adult counterpart in terms of individual‐level biomarkers. A meta‐analysis showed that interindividual behavioral variability was primarily related to the connectome of the association cortex that had larger anatomical differences across individuals (Mueller et al., 2013). Therefore, it is possible to underestimate the correspondence between the connectome and behavior using the group‐level atlas‐based FC (Li et al., 2019). The present findings highlight the importance of accounting for the variability in cortical functional region boundaries in adolescents when screening for FC biomarkers of AOS symptom burden. Connectome‐based biomarkers can reveal AOS positive symptom‐related circuits; moreover, individual‐specific FC biomarkers map symptom‐related circuits onto the individual brain, providing a basis for more precise diagnosis and treatment in AOS.

The severity of AOS positive symptoms was linked to FPN‐based connections. The FPN is responsible for high‐order executive control functions in working memory (Coull, Frith, Frackowiak, & Grasby, 1996), object orientation (Corbetta, Kincade, Ollinger, McAvoy, & Shulman, 2000), and attention (Corbetta & Shulman, 2002; Kanwisher & Wojciulik, 2000; Kastner & Ungerleider, 2000), and continuously updates and maintains changes to attended stimuli in order to adapt to the environment (Duncan, 2010). During adolescence, frontoparietal regions undergo structural fine‐tuning and maturation (Gogtay et al., 2004; Lenroot & Giedd, 2006). However, dysregulation of these processes can lead to frontoparietal gray matter abnormal loss (Burke, Androutsos, Jogia, Byrne, & Frangou, 2008) and disruption of FC (Kyriakopoulos et al., 2012; White, Schmidt, Kim, & Calhoun, 2011). Impaired inhibitory control of the FPN may underlie the positive symptoms of schizophrenia—for example, disordered self‐monitoring of internal speech (Rubio Gomez et al., 2010). Our findings revealed an association between the FPN dysconnectivity and AOS positive symptoms, suggesting the critical role of FPN in AOS positive symptom manifestation. Moreover, the novel individual‐level findings indicate the possibility to develop personalized target‐therapy schemes of AOS positive symptoms in terms of FPN‐based FC biomarkers.

In support of the neurodevelopmental hypothesis of schizophrenia (Murray & Lewis, 1987), we observed that AOS patients showed atypical age‐related alterations of FPN–SAL connection, which made significant contribution to the prediction of AOS positive symptoms. In accordance with evidence for aberrant development of frontoparietal regions in AOS (White et al., 2011), the current findings further reveal its association with the manifestation positive symptoms of AOS. Specifically, positive connections between dlPFC‐based frontopaterial subnetwork (i.e., CEN) and SAL increased with age in HC subjects, but not in AOS patients. The dlPFC is one of the last regions to fully mature at the end of adolescence, with synaptic pruning and myelination occurring in parallel (Gogtay et al., 2004). Abnormal age‐related maturation has been reported in the dlPFC‐based network connectivity of AOS (Kyriakopoulos et al., 2012). Furthermore, during adolescence, reinforcement of network‐level connectivity between CEN and SAL may reflect an increased capacity for high‐order cognitive control, which governs appropriate responses to salient external stimuli and internal events (Duan et al., 2019; Menon, 2011). Accordingly, altered maturation of CEN–SAL connectivity during adolescent could underlie the delusions and hallucinations experienced by in AOS patients (Sommer et al., 2008), which might shed some new insights into early diagnosis or treatment of schizophrenia.

This study had several limitations. First, the sample size was relatively small, which may have limited the statistical power of the prediction model of the brain–behavior relationship; therefore, our model requires validation in a larger sample size of antipsychotic‐naïve, first‐episode AOS patients. Second, the reliability of our findings on age‐related development was limited by the lack of a longitudinal study, as cross‐sectional developmental trajectories could be due to interindividual differences rather than the effects of age. Third, our model could not estimate negative symptoms for two possible reasons: some ROIs were excluded if they were absent in any participant so that we could identify consensus ROIs across all participants; this may have discounted potentially important functional information associated with negative symptoms. However, the influence of such an omission was reduced by the fact that the remaining 79 consensus ROIs (covered area ratio = 76.89 ± 1.39%) included all 18 cortical functional networks. Alternatively, subcortical connectivity was not included in our prediction model due to the lack of a reliable technique for mapping individual‐level subcortical regions. Adapting a method of individual‐level functional parcellation to subcortical regions is recommended in view of their involvement in psychiatric disorders. Finally, the group‐level functional network atlas which we used in the iterative parcellation algorithm was based on the adult population, which may disturb individual‐specific cortical parcellation results of adolescent subjects. For example, this limitation may partly account for lower covered area ratio of homologous ROIs compared with previously studies (Brennan et al., 2019; Wang et al., 2018). Future studies based on a pediatric‐based functional parcellation atlas are needed to validate current results.

## 5 CONCLUSION

Using a novel individual‐specific cortical parcellation approach, we identified reproducible FPN‐based cortical network connection biomarkers underlying positive AOS symptoms, which reflected aberrant age‐related alterations consistent with the neurodevelopmental hypothesis of schizophrenia. Our findings provide insight into the neural correlates of AOS positive symptom manifestation and suggest that individual‐level FC biomarkers can lead to more precise diagnosis and personalized target‐therapy schemes for this disorders.

## CONFLICT OF INTEREST

The authors declare no conflict of interest.

## AUTHOR CONTRIBUTIONS

**Yun‐Shuang Fan**, **Huafu Chen**, and **Hesheng Liu**: Contributed to the conception and design of the work. **Yong Xu**, **Jingping Zhao**, Peng Yue and **Yan Zhang**: Contributed to the acquisition and interpretation of data for the work. **Yun‐Shuang Fan**, **Liang Li**, **Haoru Li**, **Jing Guo**, and **Meiling Li**: Contributed to the analysis of data. **Yun‐Shuang Fan**, **Xiaonan Guo**, **Shaoqiang Han**, and **Siqi Yang**: Drafted the work. **Wei Liao**, **Qian Cui**, and **Xujun Duan**: Revised it critically for important intellectual content. All authors performed final approval of the version to be published, and agreed to be accountable for all aspects of the work in ensuring that questions related to the accuracy or integrity of any part of the work are appropriately investigated and resolved. All authors have approved this manuscript.

## Supporting information


**Appendix** **S1:** Supporting information

## Data Availability

Data sharing is not applicable to this article as no new data were created or analyzed in this study.
