# Supplementary material for: Individual‐specific functional connectome biomarkers predict schizophrenia positive symptoms during adolescent brain maturation
Source: Hum Brain Mapp. 2020 Dec 2;42(5):1475–84. doi: 10.1002/hbm.25307 (PMC7927287; doi:10.1002/hbm.25307)
Supplement: Supplementary file 1 — Appendix S1: Supporting information [file HBM-42-1475-s001.doc]

**Supplementary Material**

**Individual-specific functional connectome biomarkers predict schizophrenia positive symptoms during adolescent brain maturation**

Running title: Individualized FC biomarkers in schizophrenia

**Yun-Shuang Fan^1^, Liang Li^1^, Yue Peng^2^, Haoru Li^1^, Jing Guo^1^, Meiling Li^1,3^, Siqi Yang^1^, Meng Yao^1^, Jingping Zhao^5^, Hesheng Liu^3^, Wei Liao^1^, Xiaonan Guo^1^, Shaoqiang Han^1^, Qian Cui^1^, Xujun Duan^1^, Yong Xu^*4^, Yan Zhang^*2^, Huafu Chen^*1^**

^1^ The Clinical Hospital of Chengdu Brain Science Institute, MOE Key Lab for Neuroinformation, School of life Science and technology, University of Electronic Science and Technology of China, Chengdu, China; ^2^ Department of Psychiatry, the Second Affiliated Hospital of Xinxiang Medical University, Xinxiang, China; ^3^ Athinoula A. Martinos Center for Biomedical Imaging, Department of Radiology, Massachusetts General Hospital, Harvard Medical School, Charlestown, MA, USA; ^4^ Department of Psychiatry, First Hospital/First Clinical Medical College of Shanxi Medical University, Taiyuan, China; ^5^ Institute of Mental Health, the Second Xiangya Hospital, Central South University, Changsha, China.

*Corresponding author: Huafu Chen, The Clinical Hospital of Chengdu Brain Science Institute, MOE Key Laboratory for Neuroinformation, University of Electronic Science and Technology of China, Chengdu 610054, P.R. China. E-mail: chenhf@uestc.edu.cn; Yan Zhang, Department of Psychiatry, the Second Affiliated Hospital of Xinxiang Medical University, Xinxiang, China. E-mail: zhangyan800102@126.com; Yong Xu, Department of Psychiatry, First Hospital/First Clinical Medical College of Shanxi Medical University, Taiyuan, China. E-mail: xuyongsmu@vip.163.com.

**Supplementary 1.**

Given that brain structure and function vary with age, normalizing the adolescent data by using an adult template may disturb the results. Therefore, the T1-weighted anatomical images were normalized into an NIH-pediatric template (asymmetric MINC1 templates of post puberty; http://www.bic.mni.mcgill.ca/ServicesAtlases/NIHP

D-obj1) to find out whether the results can be affected by the normalization template. Subsequent data preprocessing and further analyses were identical with the primary analyses.

In the validation results, 80 homologous individual-specific ROIs were identified, parcellation map of which was approximately the same as the primary 79-ROIs map (Fig. S1). Furthermore, group-averaged functional connectome matrices among individualized ROIs across all subjects were also similar between the two results (Fig. S2). More importantly, PANSS positive scores of AOS could also be predicted by a set of individualized functional connections in the validation results (r = 0.48, P = 0.01; permutation test). Therefore, the validation results supported our main results, indicating that the template we used in normalization did not significantly affect our results.


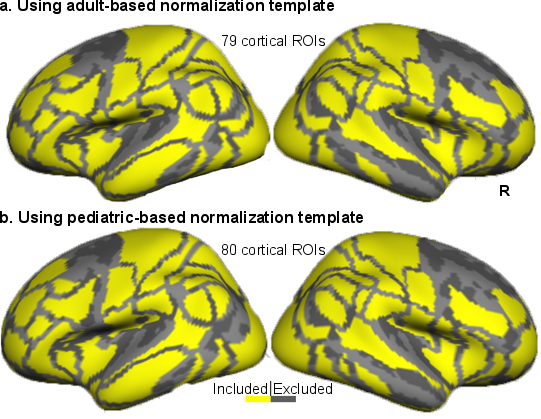


**Fig. S1. Consensus ROI maps were similar.**


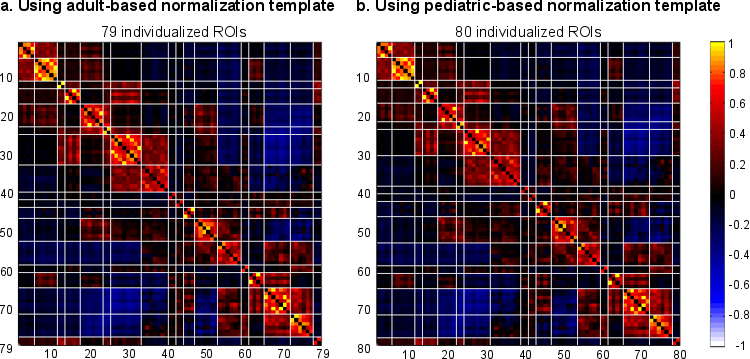


**Fig. S2. Two group-averaged FC matrices among individualized ROIs in the primary results and validation results.**

**Supplementary 2.**

**Replication cohort**

**Participants**

Twenty-nine antipsychotic-naïve first-episode adolescent-onset schizophrenia (AOS) patients were recruited from outpatient treatment centers of the First Hospital of ShanXi Medical University. Patients were diagnosed based on the consensus by two experienced psychiatrists using the Structured Clinical Interview for Diagnostic and Statistical Manual of Mental Disorders, Fourth Edition. Exclusion criteria for all subjects were as follows: neurological or other psychiatric diseases; current (within the last 12 months) substance use; neurological MRI anomalies; or any electronic or metal implants. The Positive and Negative Syndrome Scale (PANSS) was used to assess the severity of psychotic symptoms in AOS patients. All the patients were right-handed Chinese Han people aged between 12 and 18 years. This study was approved by the Ethics Committe of the First Hospital of ShanXi Medical University, and written consent was obtained from all participants and their parents.

**Data acquisition**

Imaging data of the replication cohort were collected using a 3 Tesla MRI scanner (MAGNETOM Verio; Siemens, Germany) around one year after the primary cohort acquisition. The primary imaging data were collected based on the software syngo MR B17, while the replication data were collected based on an upgraded software version, i.e., syngo MR B19. Participants were instructed to stay awake with their eyes closed during the scan. Functional images were acquired as an echo-planar imaging sequence with the following parameters: repetition time (TR) = 2500 ms; echo time (TE) = 30 ms; matrix = 64 × 64, 32 axial slices; slice thickness = 3 mm, 1 mm gap; flip angle = 90°; field of view = 240 × 240 mm^2^; voxel size = 3.75 × 3.75 × 4 mm^3^; and 212 volumes. T1-weighted anatomical images were acquired as a three-dimensional fast-spoiled gradient-echo sequence with the following parameters: TR = 2300 ms; TE = 2.95 ms; matrix = 240 × 256, 160 axial slices; slice thickness = 1.2 mm, no gap; flip angle = 9°; field of view = 225 × 240 mm^2^; and voxel size = 0.9375 × 0.9375 ×1.2 mm^3^.

**Table S1.** Demographic and Clinical Characteristics.

| **Characteristic** | **Primary**  **AOSs**  (n = 30) | **Replication AOSs**  (n = 25) | **Group comparisons** | |
| --- | --- | --- | --- | --- |
|  |  |  | Statistic values | P values |
| Sex (male/ female) | 15/ 15 | 10/ 15 | 0.55 ^a^ | 0.46 |
| Age (years) | 15.10 ± 0.32 | 15.36 ± 0.26 | 0.62 ^b^ | 0.54 |
| PANSS scores |  |  |  |  |
| Total scores | 75.17 ± 2.00 | 78.64 ± 3.53 | 0.89 ^b^ | 0.37 |
| General scores | 34.13 ± 1.25 | 37.56 ± 1.65 | 1.68 ^b^ | 0.09 |
| Positive scores | 20.33 ± 1.08 | 18.08 ± 0.96 | 1.53 ^b^ | 0.13 |
| Negative scores | 20.70 ± 1.68 | 17.64 ± 1.30 | 1.39 ^b^ | 0.17 |

Mean ± SEM; AOSs, adolescent-onset schizophrenia patients; PANSS, Positive and Negative Symptom Scale.

^a^ The χ^2^ value for gender distribution was obtained by chi-square test.

^b^ The T values were obtained by two sample t-test.

**Supplementary 3.**

**Table S2.** Cortical functional network atlas.

| **Number** | **Name** | **Cerebral cortical regions** | **Network** |
| --- | --- | --- | --- |
| 1 | Visual A | Lateral V1 | VIS |
| 2 | Visual B | Medial V1 | VIS |
| 3 | Somatomotor A  (Foot) | Foot sensorimotor | MOT |
| 4 | Somatomotor B  (Tongue) | Central sulcus, secondary somatosensory, insula | MOT |
| 5 | Somatomotor C (Auditory) | Temporal | MOT |
| 6 | Somatomotor D (Hand) | Hand sensorimotor | MOT |
| 7 | Dorsal attention A | Posterior temporal, postcentral gyrus, frontal eye fields, precentral ventral frontal | ATN |
| 8 | Dorsal attention B | Posterior temporal-occipital, superior parietal, inferior parietal-occipital | ATN |
| 9 | Ventral attention | Parietal operculum, medial parietal, medial frontal, precentral central frontal, insula, temporal, precentral frontal, posterior temporal | SAL |
| 10 | Control A | Medial posterior prefrontal, ventral prefrontal, cingulate sulcus, inferior parietal, lateral prefrontal | FPN |
| 11 | Control B | Precuneus, posterior cingulate | FPN |
| 12 | Control C  (Central executive) | intraparietal sulcus, lateral prefrontal, posterior temporal, dorsal prefrontal, cingulate, orbitofrontal, medial posterior prefrontal, lateral posterior prefrontal | FPN |
| 13 | Control D | Lateral posterior prefrontal, lateral anterior prefrontal, inferior parietal, temporal, medial posterior prefrontal | FPN |
| 14 | Limbic A | Temporal pole | LMB |
| 15 | Limbic B | Orbitofrontal | LMB |
| 16 | Default A | Retrosplenial, parahippocampal complex, ventral inferior parietal | DMN |
| 17 | Default B | Medial prefrontal, posterior inferior parietal, posterior cingulate, dorsal prefrontal, orbitofrontal, temporal | DMN |
| 18 | Default C | Dorsal prefrontal, temporal, anterior inferior parietal | DMN |

Note: VIS, the visual network; MOT, the sensorimotor network; ATN, the attention network; SAL, the salience network; FPN, the frontoparietal control network; LMB, the limbic network; DMN, the default model network.


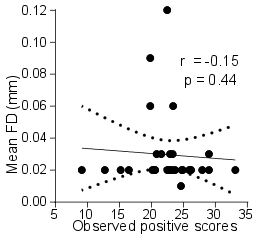


**Fig. S3. The contribution of head motion in the prediction model.**

**Supplementary 4.**

Inter-individual variability in size and position of the homologous ROIs was quantified, separately . Specifically, the size of a ROI was estimated as the number of vertices within the region; its standard deviation across subjects was used to evaluate inter-individual variability in ROI size. Regarding the location of a ROI, the average geodesic distance among the ROI centers across subjects was calculated to evaluate inter-individual variability. Ultimately, the raw inter-individual variability was normalized to 0-1 range. More importantly, one-sample t-tests were carried out for inter-individual variability in ROI size and position across all ROIs to estimate statistical significance. Results of statistical analyses suggested that the sizes (t_(78)_ = 14.20, p < 0.0001) and locations (t_(78)_ = 14.56, p < 0.0001) of the ROIs varied significantly among individuals.


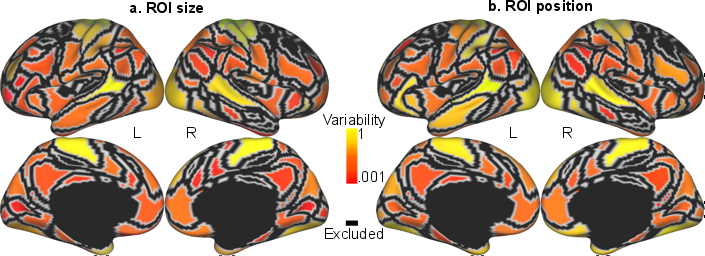


**Fig. S4. Inter-individual variability in size (a) and position (b) of seventy-nine consensus ROIs.**

**Supplementary 5.**

Compared with the original three subscales, a five-factor model (including the positive symptom factor, the negative symptom factor, the depression/anxiety factor, the disorganized thought factor, and the excitement factor) may better capture PANSS structure in schizophrenia patients. Most importantly, these factors (especially the disorganized thought factor) have been shown to be associated with specific cognitive profiles . Therefore, we added validation analyses to explore individual-level biomarkers underlying these five symptom dimensions by using two common five-factor models (See Table S3), i.e., the Marder model and the Lindenmayer model . Validation results of the Marder model: positive scores (r = 0.45, P = 0.02; permutation test) and depression/anxiety scores (r = 0.43, P = 0.03) could be predicted by individual-level FC; neither of negative scores (r = 0.11, P = 0.19), disorganized thought scores (r = −0.01, P = 0.61), and excitement scores (r = −0.43, P = 0.12) could be predicted in AOS patients. Validation results of the Lindenmayer model: only positive scores could be estimated in AOS patients (r = 0.43, P = 0.03); others including negative scores (r = 0.08, P = 0.23), depression/anxiety scores (r = 0.33, P = 0.06), disorganized thought scores (r = 31, P = 0.07), and excitement scores (r = −0.06, P = 0.58) could not.

Consistent with our primary results, positive symptom factors in whether the Marder model or the Lindenmayer model could be predicted by FPN-based individual-level FC (Fig. S5 & S6), and neither of negative symptom factors could be estimated in AOS patients. Additionally, Marder depression scores could be predicted by a set of DMN-based connections (Fig. S7), and Lindenmayer depression scores could also be approximately predicted. Whereas the two five-factor models were generally similar, the Marder model may perform slightly better on characterizing PANSS data. These results conjointly verified our primary results of the original three-subscale model. Moreover, it revealed individual-level FC biomarkers underlying depression/ anxiety symptoms in AOS.

**Table S3.** Two five-factor models of the PANSS (AOS patients = 30).

| **Factor** | **The Marder model** | **The Lindenmayer model** |
| --- | --- | --- |
| Positive symptom | 22.40 ± 0.78 | 11.07 ± 0.58 |
| Negative symptom | 21.13 ± 1.79 | 18.77 ± 1.57 |
| Depression/anxiety | 4.67 ± 0.26 | 8.10 ± 0.53 |
| Disorganized thought | 17.60 ± 0.85 | 12.20 ± 0.65 |
| Excitement | 8.13 ± 0.95 | 7.40 ± 0.74 |

Mean ± SEM.


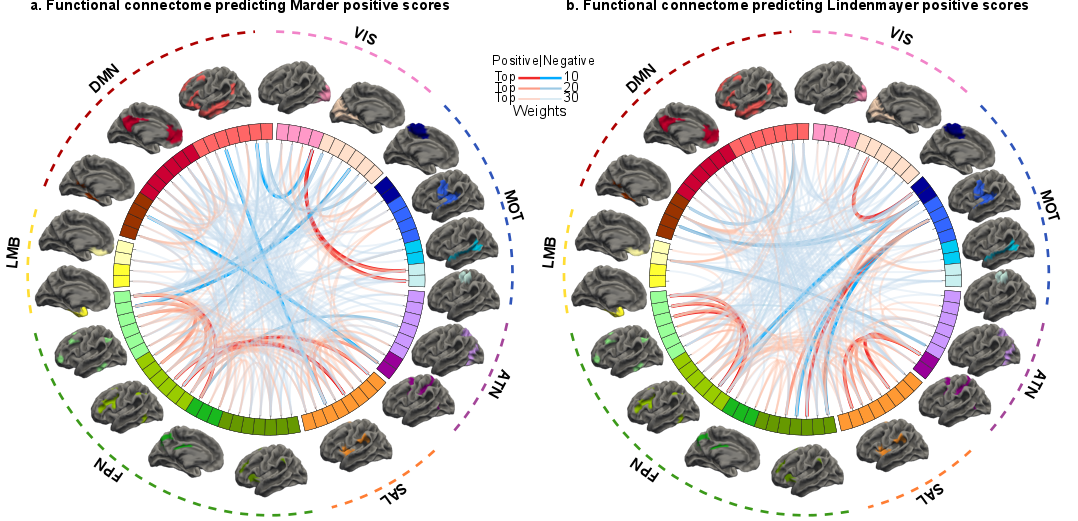


**Fig. S5. Individual-level FC biomarkers underlying positive symptom scores of the Marder model and the Lindenmayer model.**


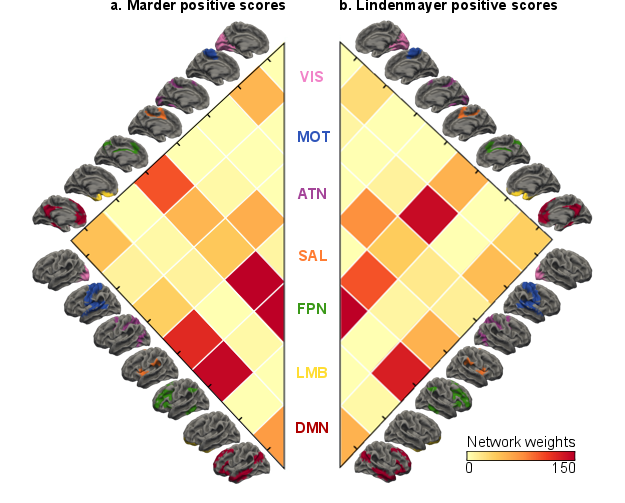


**Fig. S6. Network-level predictive weights for Marder and Lindenmayer positive symptom scores.**


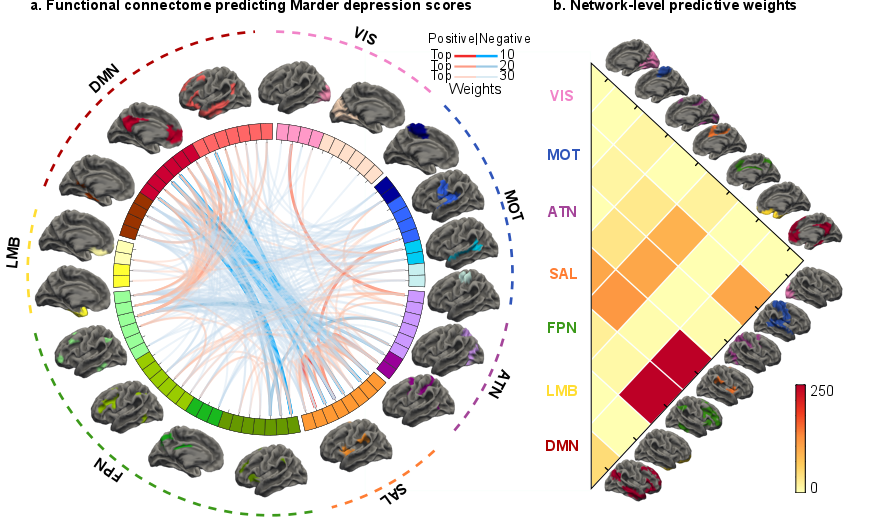


**Fig. S7. Individual-level FC biomarkers underlying Marder depression symptom scores.**

**References**

Li, M., Wang, D., Ren, J., Langs, G., Stoecklein, S., Brennan, B. P., . . . Liu, H. (2019). Performing group-level functional image analyses based on homologous functional regions mapped in individuals. *PLoS Biol, 17*(3), e2007032. doi: 10.1371/journal.pbio.2007032

Lindenmayer, J.-P., Bernstein-Hyman, R., & Grochowski, S. (1994). Five-factor model of schizophrenia: Initial validation. *Journal of Nervous and Mental Disease, 182*(11), 631-638. doi: 10.1097/00005053-199411000-00006

Marder, S. R., Davis, J. M., & Chouinard, G. (1997). The effects of risperidone on the five dimensions of schizophrenia derived by factor analysis: Combined results of the North American trials. *The Journal of clinical psychiatry, 58*(12), 538-546. doi: 10.4088/JCP.v58n1205

Wallwork, R. S., Fortgang, R., Hashimoto, R., Weinberger, D. R., & Dickinson, D. (2012). Searching for a consensus five-factor model of the Positive and Negative Syndrome Scale for schizophrenia. *Schizophr Res, 137*(1-3), 246-250. doi: 10.1016/j.schres.2012.01.031
